# Suggestions for health information technology trials for respiratory disorders in low- and middle-income country settings: what can we learn from trials in high-income country settings?

**DOI:** 10.1038/npjpcrm.2015.45

**Published:** 2015-07-09

**Authors:** Niels H Chavannes, Robert S Du Puy, Chunxue Bai

**Affiliations:** 1 Leiden University Medical Center, Department of Public Health and Primary Care, Leiden, Netherlands; 2 Department of Pulmonary Medicine, Fudan University Zhongshan Hospital, Shanghai Respiratory Research Institute, Shanghai, China

## Abstract

Health Information Technology (HIT) is sometimes seen as a silver bullet for human resource, medical and economic challenges facing health systems. The evidence supporting widespread use of HIT is, however, still patchy and inconsistent. In this Perspective piece, we seek to interpret and draw key lessons from a selection of illustrative trials in developed countries with robust health-care settings in respiratory medicine that failed to demonstrate effectiveness, and offer suggestions to maximise the chances of success in subsequent HIT deployments. Particularly low- and middle-income countries, with relatively weak health infrastructures and limited health care, propose considerable room for improvement. Early experiences of studying HIT thus far in high-income country settings suggest that this process should preferably begin with trials of low-cost, well-established technologies in patient groups with a moderate burden of disease while carefully evaluating patient safety.

## Introduction

Futurists, politicians, researchers and health-care insurers are excited about the grand promises of health information technology (HIT; also sometimes known as medical informatics or eHealth), with projected annual savings ranging up to $81 billion in the United States from a single HIT intervention alone.^1^ Often, HIT is promoted as a silver bullet for the human resource, medical and economic crises in health care, which are driven by the worldwide steep increase in patients with long-term conditions and associated costs.^2^ The field of respiratory medicine, with an increasing prevalence of Chronic Obstructive Pulmonary Disease (COPD) and persistent burden of disease of asthma, tuberculosis and lung-cancer,^3^ could offer a fruitful setting for future HIT implementations. The scientific evidence to support the use of HIT applications on a large scale is, however, still patchy and inconsistent. An analysis of 108 systematic reviews on the impact of electronic health records, computerised physician order entry, computerised decision support systems and ePrescribing technologies revealed that the bold claims of clinical effectiveness sometimes made by policymakers and vendors are seldom supported by sound evidence.^4^ A subsequent follow-on systematic overview of telehealth interventions came to a similar conclusion.^5^ Most of these HIT evaluations have been undertaken in high-income country settings, and an increasing body of literature reveals a discrepancy between the anticipated impact and the demonstrated effectiveness of HIT interventions.

In this Perspective piece, we seek to interpret and draw key lessons from a selection of earlier high-quality illustrative large randomised clinical trials in developed countries with robust health-care settings that failed to demonstrate effectiveness, with a view to offering suggestions to maximise the chances of success in subsequent HIT deployments. In particular, there is considerable interest and activity in implementing HIT in many low- and middle-income countries (LMICs) and it is crucial that such implementations draw on lessons from previous attempts in high-income country settings.

## Identification of papers

We searched PubMed for recent trials of HIT interventions for respiratory and allergic disorders, preferably undertaken in primary care contexts. Potentially relevant papers were extracted, referenced articles were obtained and a selection of illustrative trials from all identified papers was made.

## Illustrative experiences in high-income settings

### Possible erroneous algorithms

In 2012 a study on the results of HIT was published, demonstrating not only an inconsistency in anticipated and empirical effectiveness but unexpected adverse results as well.^6^ To investigate a possible effect on hospital admissions or emergency visits in patients with several chronic (including respiratory) conditions, an individually randomised controlled trial was performed in the United States, comparing daily telemonitoring (including biometry, symptom registration and video consultation by Internet) with usual care during 1 year. This well-conducted study among 205 elderly people with multi-morbidity (defined as a Charlson Comorbidity Index^7^ >2.9) and a high risk for hospitalisation (a score >15 on the Elder risk assessment index)^8^ did not result in significant benefit of the intervention. No statistically relevant difference was found between the intervention group of patients (63.7%) and the control group (57.3%) in the number of hospitalisations or emergency visits (*P*=0.35). However, mortality in the intervention group was considerably elevated (14.7 vs. 3.9%; *P*=0.008), which was an ominous and unexpected outcome. The authors speculated that the intensive level of care resulting from automated treatment algorithms may have led to unnecessary and sometimes invasive procedures, causing the excess mortality observed.

It is deeply worrying when three to fourfold increased mortality risks are seen in the intervention arm. If a trial investigating a novel drug resulted in such outcomes, it would no doubt come under intense regulatory scrutiny and would certainly not be approved for use, or if already in practice may be rapidly withdrawn from the worldwide market to minimise further harm. This particular intervention is at the moment no longer available in its original form.

### Insufficient considerations of sociotechnical implications

The reasons for unsuccessful HIT interventions are complex and not yet fully understood. Testing of relatively immature technologies, a failure to adequately attend to sociotechnical considerations, the execution of relatively small trials with large postulated clinical effect sizes, and limited headroom for improvement in societies with well-developed health-care infrastructures have been proposed as possible explanations.^4,5^ These arguments are illustrated by a recent small pilot randomised controlled trial (RCT) in the Netherlands in which telemedicine care, electronic communication through phone calls with patients to serve as an alternative or extension to traditional outpatient visits, was compared with traditional care in stable COPD patients.^9^ Symptom deterioration measured on the St George’s Respiratory Questionnaire was significantly (*P*=0.02) higher in the treatment arm (6.1 points) compared with the controls (−2.8 points). In addition, financial and time resource use in both primary and secondary care for COPD over 6 months of follow-up had increased in the treatment arm, with significantly more visits to the pulmonologist and, although not statistically significant, more exacerbations compared with the controls. In the discussion section the authors argued, in line with earlier hypotheses from COPD HIT interventions,^10,11^ that telemedicine interventions alone in well-developed (primary) health-care systems intrude on an already optimised strategy of care and might even reduce care quality. Furthermore, the HIT intervention may have resulted in a heightened awareness of their illness, decreasing their natural acceptance and coping capabilities, deteriorating the perceived quality-of-life. In primary care, where care not only focuses on disease parameters but on general well-being in a longitudinal context as well, HIT may not be the Holy Grail for all patients with chronic disease, and could unintentionally even stunt further development of primary care health systems if applied incorrectly.

### Limited headroom for improvement

Recent evidence from a large multicentre HIT randomised controlled trial, which attempted to address the earlier identified reasons for unsuccessful implementation, offering the same structured clinical and educational intervention to two groups of participants did not demonstrate better results when the only difference was the use of mobile phone technology in one treatment arm.^12^ The carefully set-up trial assessed the clinical and cost-effectiveness of mobile phone-supported self-monitoring of 288 poorly controlled asthma patients compared with paper-based monitoring, by monitoring of lung function, self-efficacy and symptoms using electronic questionnaires and providing instantaneous feedback. Over a 6-month follow-up period, no significant differences were found in the two groups regarding asthma control (mean difference in change −0.02, 95% confidence interval (95%CI) −0.23–0.19) or self-efficacy (mean difference in change 2.0, 95%CI −0.3–4.2). Moreover, the added costs of the mobile phone technology intervention rendered the intervention not cost-effective.^12^

## Interpretation

### Scope for improvement

With the emergence of reports on ineffective HIT implementations, concerns may arise as to whether HIT is an effective tool in health care at all. In our opinion, this reflects a naïve position, as HIT is an umbrella term that includes within it a very broad array of interventions and because HIT invariably needs to be understood within a wider organisational and sociotechnical framework. Further, as research into HIT progresses, we are developing a more sophisticated appreciation of the contexts in which HIT is likely to be of particular benefit. As technology matures and investigators design trials with respect to realistic and likely effect sizes, the sensitivity to detect smaller effects increases. Moreover, we believe that the room for improvement in both patient health and health infrastructures proposes considerable promise for settings with weak health infrastructures and limited health care. This is illustrated by the fact that in a recent HIT trial on food-related quality-of-life improvement using 24-hour telephone access to specialist clinical advice in children with food-allergy-triggered anaphylaxis, Kelleher *et al* demonstrated that, in settings with substantial room for clinical improvement, technology can help achieve major improvements in outcomes.^13^

### Low- and middle-income counties

LMICs, often with suboptimal local standards of primary care and general poor health with ample headroom for development, are therefore in many ways ideally suited for carefully developed HIT interventions. Both in developed and in developing countries, long-term respiratory conditions increasingly contribute to the burden of disease, making this area of medicine particularly interesting to benefit from future HIT trials. Considering that HIT development and evaluation in developing countries seems to follow the course of development in developed countries,^14^ we believe that to improve the chances of successful implementations in LMICs it is essential to learn from earlier experiences of HIT interventions in high-income settings and to avoid repeating earlier identified mistakes.

### Key lessons

If we are to maximise the benefits associated with HIT interventions while minimising risks in LMICs, we need to establish (1) the effective components^15^ and (2) the actual patient group likely to benefit, before implementing often highly complex HIT applications. Often, simple solutions, carefully tailored to local needs, offer the greatest chance for success. Therefore, we propose there is considerable scope to learn from the early experiences in high-income country settings. Key lessons include the following:

- Importance of not seeing HIT as a silver bullet
- Preferentially considering low-tech, low-cost interventions
- Careful theorising about likely causal pathways and hence patient groups or health-care systems most likely to benefit/least likely to be harmed
- Recognising the interplay of HIT and sociotechnical considerations
- The need for realistic assessments of likely benefit and hence large sample sizes (patient/years of follow-up) to ensure adequate statistical power
- Recognition that HIT may prove ineffective/result in adverse effects and hence the need for independent, rigorous evaluations (preferably using RCT designs and systematic reviews)
- Accompanying process, qualitative and health economic evaluations
- Avoiding premature roll-out of HIT ‘solutions’ until there is a secure evidence base.

The studies discussed in this Perspective paper suggest that, in complex disease, personal attention and commitment cannot be simply replaced by automated treatment algorithms. Probably, both patients and health-care providers sometimes overly rely on technology, inadvertently changing their testing and referral behaviour, possibly leading to unwanted effects in both intervention and control groups. Possibly, policymakers, researchers and clinicians take too large strides when it comes down to HIT applications. Similar to the introduction of novel drugs, there is a need to systematically evaluate complex HIT interventions before large-scale roll-out, without hampering innovation.^16^ We must be able to simultaneously evaluate HIT interventions while they are being designed, developed and deployed.^17^ Although HIT could be a promising area in health care, we urge future researchers, physicians and patients to treat and research HIT like any other intervention. HIT, at least in primary care, is a possible extension to diagnostic and therapeutic availabilities of health care that needs to be thoroughly researched for effectivity, cost and safety.

### Careful introduction

As a first step for introducing HIT interventions, particularly in LMICs, we propose low-cost, low-tech HIT applications in patient groups with a moderate burden of disease, as sufficient symptoms will be present (offering room for improvement) while safety is not commonly an issue yet (as opposed to severe disease). Crucially, direct personal contact with a dedicated and experienced health-care provider should be provided as backup. Alternatively, one could consider simple yet promising interventions such as mobile phone text-message reminders, depending on highly variable local needs. Recently, several studies from other fields of medicine evaluating HIT in LMICs that comply with the above-mentioned proposed key lessons have demonstrated good clinical results or improvement of health-care systems in primary care. For example, in a large RCT on text-messaging, 537 patients with impaired glucose tolerance from South India were randomly assigned either to the mobile phone messaging intervention with frequent automated starting cues for physical activity, information on diabetes and lifestyle, and motivational texts (*n*=271) or to standard care (*n*=266).^18^ The cumulative incidence of type 2 diabetes was lower in those who received mobile phone messages than in controls over a mean follow-up time of 20.2 months: 50 (18%) participants in the intervention group developed type 2 diabetes compared with 73 (27%) in the control group (hazard ratio 0.64, 95%CI 0.45–0.92; *P*=0.015). The number needed to treat to prevent one case of type 2 diabetes was 11 (95%CI 6–55). Moreover, a large cluster RCT of simple mobile phone text-message reminders^19^ showed that Kenyan health workers’ adherence to malaria treatment guidelines could be improved by 23.7% points (95%CI 7.6–40.0; *P*=0.004) immediately after intervention and by 24.5% points (8.1–41.0; *P*=0.003) 6 months later. Hence, future trials could start in patient groups with low-to-moderate burden of disease, eventually scaling up to implementing interventions in highly complex patient groups with less personal guidance when more robust scientific evidence for effectiveness and safety has been obtained.

### Future HIT in respiratory primary care

With increasing global attention on HIT interventions in low-resource settings, such as the calls for innovative health technologies for low-resource settings issued annually by the World Health Organization, specifically on pneumonia and chronic respiratory diseases (http://www.who.int/medical_devices/innovation/call_2014/en/index4.html), and the increased feasibility through innovative research and development by stakeholders, such as the Satellife personal digital assistant (PDA) handheld devices (PDAs issued to African medical professionals loaded with medical reference tools and texts, field surveys and guides for diagnosing diseases) powered by supplementary on-site solar stations (http://www.satellife.org/), research possibilities in LMICs are expanding. Several ongoing studies from LMICs offer good implementation prospects by offering relatively simple and safe HIT interventions in settings with room for improvement. For example, the Ecompliance programme executed in India, Cambodia, Uganda, the Dominican Republic and Kenya is evaluating whether directly observed treatment regimens for tuberculosis can be improved by recording finger-print scans of patients on a biometric attendance terminal after every dose ingestion, and offer encouragement and coaching to patients found absent from therapy appointments. Initial qualitative results are hopeful; however, future quantitative analysis is needed to assess the clinical benefits and cost-effectiveness of the intervention.^20^ Another example is the well-designed MIOTIC Study, a multicentre RCT that evaluated long-term efficacy of mobile phone-based internet-of-things HIT in the management of 600 Chinese patients with stable COPD to overcome limited medical resources and increased medical demand. Using mobile phones with Internet connectivity, participants can connect sensors to a cloud platform for instant measurement analysis, self-report symptomatic changes such as stress and schedule medication reminders and get instantaneous personalised advise and educational materials calculated to be tailored to a participant's needs.^21^ By including COPD patients with a moderate burden of disease, carefully and realistically assessing predicted power and follow-up time requirements, continuously evaluating effectiveness and safety and using a person-focused integral approach of the COPD patient this trial meets a comprehensive list of conditions we believe are required for successfully investigating the effectiveness of an integrated HIT intervention, and because of its design has high potential to be an effective HIT intervention.

## Conclusion

It is important that HIT interventions be formally evaluated. The evaluations undertaken in high-income country settings of respiratory conditions offer valuable insights for trials of HIT in LMICs, particularly considering that there is likely to be more headroom available to detect an effect.

Experiences of studying HIT thus far in high-income country settings suggest that this process should preferably begin with trials of low-cost, well-established technologies in those patient groups with a moderate burden of disease while carefully evaluating patient safety.

## Notes

NHC is an Associate Editor of *npj Primary Care Respiratory Medicine*, but was not involved in the commissioning or processing of this manuscript or the decision to publish it. The other authors declare no conflict of interest. All authors have contributed to writing and revision of the manuscript.
